# Identification of viral protein R of human immunodeficiency virus-1 (HIV) and interleukin-6 as risk factors for malignancies in HIV-infected individuals: A cohort study

**DOI:** 10.1371/journal.pone.0296502

**Published:** 2024-01-02

**Authors:** Akihiro Matsunaga, Naokatsu Ando, Yuko Yamagata, Mari Shimura, Hiroyuki Gatanaga, Shinichi Oka, Yukihito Ishizaka

**Affiliations:** 1 Department of Intractable Diseases, Research Institute, National Center for Global Health and Medicine, Toyama, Shinjuku, Tokyo, Japan; 2 AIDS Clinical Center, Hospital, National Center for Global Health and Medicine, Toyama, Shinjuku, Tokyo, Japan; 3 RIKEN SPring-8 Center, Koto, Sayo, Hyogo, Japan; University Hospital Zurich, SWITZERLAND

## Abstract

**Background:**

Despite effective antiretroviral therapy, patients with human immunodeficiency virus type-1 (HIV) suffer from a high frequency of malignancies, but related risk factors remain elusive. Here, we focused on blood-circulating viral protein R (Vpr) of HIV, which induces proinflammatory cytokine production and genotoxicity by exogenous functions.

**Methods and findings:**

A total 404 blood samples of HIV patients comprising of 126 patients with malignancies (tumor group) and 278 patients without malignancies (non-tumor group), each of 96 samples was first selected by one-to-one propensity score matching. By a detergent-free enzyme-linked immunosorbent assays (detection limit, 3.9 ng/mL), we detected Vpr at a higher frequency in the matched tumor group (56.3%) than in the matched non-tumor group (39.6%) (*P* = 0.030), although there was no different distribution of Vpr levels (*P* = 0.372). We also detected anti-Vpr immunoglobulin (IgG), less frequently in the tumor group compared with the tumor group (22.9% for tumor group vs. 44.8% for non-tumor group, *P* = 0.002), and the proportion of patients positive for Vpr but negative of anti-Vpr IgG was significantly higher in the tumor group than in the non-tumor group (38.6% vs. 15.6%, respectively, *P* < 0.001). Additionally, Interleukin-6 (IL-6), the levels of which were high in HIV-1 infected patients (*P* < 0.001) compared to non-HIV-infected individuals, was significantly higher in advanced cases of tumors (*P* < 0.001), and IL-6 level was correlated with Vpr in the non-tumor group (*P* = 0.010). Finally, multivariate logistic regression analysis suggested a positive link of Vpr with tumor occurrence in HIV patients (*P* = 0.002).

**Conclusion:**

Vpr and IL-6 could be risk factors of HIV-1 associated malignancies, and it would be importance to monitor these molecules for well managing people living with HIV-1.

## Introduction

Before the advent of antiretroviral therapy (ART) initiated in 1996, human immunodeficiency virus type-1 (HIV)-infected people were susceptive to a high incidence of numerous malignancies, especially AIDS-defining malignancies (ADMs) such as non-Hodgkin lymphoma (NHL), Kaposi sarcoma (KS), and invasive cervical cancer [1]. In the post-ART era, the risk of opportunistic infection and ADMs have been dramatically decreased with remarkable improvement of their mortality and quality of life [2,3]. However, lines of evidence revealed that ART did not eliminate the risk of a part of ADMs and non-AIDS defining malignancies (NADMs) that include cancers of the stomach, colon, pancreatic, anal, lung, liver, skin and prostate and Hodgkin lymphoma (HL). Strikingly, the incidence of these NADMs among people living with HIV infection (PLWH) is 1.69-fold higher compared with the general population [4–6]. Malignancies in HIV patients are characterized by more advanced tumor progression and poor prognosis, causing approximately 50% of mortality among PLWH [7,8]. It is crucially important to develop predictive and preventive methods, but both modes of NADMs and their risk factors remain elusive.

As candidate risks for NADMs, it has been proposed that the co-infection of tumor viruses, immunocompromised state, chronic inflammation (inflammatory cytokines), and viral proteins with transforming activities [9,10] are mutually involved in the tumor development. The chronic overproduction of inflammatory cytokines in response to inflammation, infection, or tissue damage can promote tumor growth and metastasis by stimulating cell proliferation, survival, angiogenesis, and invasion [11,12]. Additionally, it is particularly important to note that viral proteins such as transactivator of transcription (Tat), negative factor (Nef), glycoprotein 120 (gp120), p17, and viral protein R (Vpr) are present in patients’ blood as soluble forms and function as transforming factors [13–17]. These observations suggest that PLWH is exposed to multiple cancer risks even under ART; however, it is unclear how each molecule is involved in carcinogenesis.

Vpr is a highly conserved viral accessory protein that is packaged in the virion [18,19] and delivered into the nucleus of infected cells upon viral entry. By forced expression, Vpr shows pleiotropic functions, which include the enhancement of viral replication by transactivation of the viral promoter [20,21], the suppression of host immune responses [22,23], the activation of the oxidative stress pathway [24], DNA damage response with cell cycle arrest at the G2 phase [25–30], suppression of the repair of DNA double-strand breaks [31–33], and inhibition of telomerase activity [34]. Of note, extracellular Vpr is biologically active and induces apoptosis in astrocytes and neurons [35–37], mitochondrial oxidative stress [38,39], and DNA damage response [40,41]. We reported that extracellular Vpr has multiple functions active for induction of interleukin-6 (IL-6) production via Toll-like receptor 4 (TLR4) and MyD88 [42,43], and of retrotransposition [44]. To explore the biological relevance of Vpr i*n vivo*, we developed an enzyme-linked immunosorbent assay system for detecting Vpr (Vpr-ELISA) and reported that Vpr is present in approximately 20% of HIV patients [45], even in well-controlled PLWH with undetectable HIV RNA under ART control. Because Vpr present in blood was active in inducing retrotransposition [44], and it has been reported that Vpr activates the production of tumor necrosis factor alpha (TNFα) and Interleukin-1 beta (IL-1β) [37,46], it is plausible that Vpr itself and/or inflammatory cytokines cooperatively function as cancer risks in PLWH [47].

To prove this possibility, we measured levels of Vpr in sera of 404 PLWH with or without malignancies and analyzed the relationship between Vpr and inflammatory cytokines in relation to tumor development.

## Materials and methods

### Ethical approval

This study was performed following the national regulations and institutional policies and was approved by the ethics committees of the National Center for Global Health and Medicine (#NCGM-G-003183-00) on Mar. 8, 2019 in accordance with the Helsinki Declaration of the World Medical Association. All subjects were provided based on written informed consent.

### Study participants

We obtained 404 serum samples of HIV patients from the National Center for Global Health and Medicine (NCGM) Biobank in Japan, which were collected from the period Mar. 30, 2010 to Apr. 2, 2021. Among them, 126 samples belonged to HIV patients with malignancies (tumor group), and 278 samples belonged to HIV patients without any malignancies (non-tumor group) (Table 1). The non-tumor group had no evidence of tumor development for at least one year after the blood collection. We accessed and retrieved clinical data at the time of the sampling and the tumor-free status for at least one year from Sep. 4, 2019 to Apr. 21, 2023.

**Table 1:** Demographic and clinical characteristics of both the full cohort and the matched cohort.

HIV patients	Full cohort			Propensity matched cohort		
	Tumor	Non-tumor	*P*-value	Tumor	Non-tumor	*P*-value
	(*n* = 126)	(*n* = 278)		(*n* = 96)	(*n* = 96)	
Sex at birth						
Female	6 (5.2)	5 (6.3)	> 0.999	6 (5.2)	5 (6.3)	> 0.999
Male	90 (94.8)	91 (93.7)		90 (94.8)	91 (93.7)	
Age (years)	54 [43 – 64]	50 [47 – 57]	0.289	54 [43 – 64]	50 [47 – 57]	0.219
CD4+ T-cells (cells/μL)	435.0 [277.3–602.3]	450.0 [297.5–573.5]	< 0.001	435.0 [277.3–602.3]	450.0 [297.5–573.5]	0.908
CD8+ T-cells (cells/μL)	650.0 [465.5–914.3]	700.5 [483.8–935.8]	0.159	650.0 [465.5–914.3]	700.5 [483.8–935.8]	0.773
CD4/CD8 ratio	0.661 [0.389–1.027]	0.660 [0.390–1.000]	< 0.001	0.661 [0.389–1.027]	0.660 [0.390–1.000]	0.832
Viremia						
HIV	19 (19.8)	22 (22.9)	< 0.001	19 (19.8)	22 (22.9)	0.725
HBV	12 (12.5)	6 (6.3)	0.0502	12 (12.5)	6 (6.3)	0.215
HCV	11 (11.5)	4 (4.2)	0.0010	11 (11.5)	4 (4.2)	0.104
Tumor Stages^a^						
local	53 (42.1)			53 (42.1)		
advanced	72 (57.1)			72 (57.1)		

Data are *n* (%) or median [interquartile range].

a, there is a missing information on one case.

Fisher’s exact test was used to test for differences in categorical variables.

Mann–Whitney *U* test was used to test for differences in continuous variables for full cohort.

Wilcoxon matched-pairs rank test was used to test for differences in continuous variables for propensity matched cohort.

The tumor group consisted of 51 patients with ADMs and 75 with NADMs. The ADM patients included 28 cases with NHL, 22 with KS, and 1 with cervical cancer, while the NADM patients included 12 cases with colon cancer, 8 with anal cancer, 7 with stomach cancer, 5 with pancreatic cancer, 4 with liver cancer, 4 with oral cancer, 3 with prostate cancer, 3 with lung cancer, 3 with skin cancer, 7 with HL, and 19 with other cancers. The serum samples were aliquoted, frozen, and stored at –80°C. The demographic and clinical characteristics of all participants were obtained using routine clinical tests and included age, sex, blood CD4+ and CD8+ T-cell counts (cells/μl), plasma HIV RNA, hepatitis B virus (HBV) antigen, and hepatitis C virus (HCV) antibody. The tumor stages, local and advanced, were determined by professional physicians. The advanced stage means that the tumor has spread to the tissues around primary lesion or the other organs of the body.

### Vpr-ELISA

The Vpr-ELISA was performed as previously reported [45]. Briefly, microtiter plates pre-coated with polyclonal antibodies against recombinant Vpr (rVpr) were blocked with a buffer comprising 1% BlockAce (Megmilk Snow Brand, Tokyo, Japan) diluted in phosphate-buffered saline, pH 7.4 (PBS). Then, 10 μL serum or standard rVpr was incubated at 37°C for 1 h with 40 μL of 0.4% BlockAce/PBS. After washing with PBS, 50 μL of 5 ng/μL B34 anti-Vpr monoclonal antibody was applied and incubated at 37°C for 1 h, followed by incubation with a horseradish peroxidase (HRP)-conjugated anti-penta-His antibody (Thermo Fisher Scientific, Waltham, MA). 3,3′,5,5′-Tetramethylbenzidine (TMB) substrate was added for development, and the signal was measured after 30 min at a wavelength of 450 nm using a microplate reader (Bio-Rad, Hercules, CA). By optimizing the incubation temperature (37.0°C) and pH condition (pH 7.40) of the solutions, we tried improving the signal-to-background ratio and successfully increased the detection limit to 3.9 from 39 ng/mL compared with the previous study. All samples were measured in triplicate.

### Anti-Vpr IgG ELISA

According to the reported procedures [48], rVpr was produced by a wheat germ cell-free protein expression system (CellFree Sciences, Ehime, Japan) and by using strep-tagII (IBA Lifesciences, Göttingen, Germany) and His-tag (Qiagen, Hilden, Germany) affinity purification methods. Microtiter plates (Thermo Fisher Scientific) were coated with 50 μL of 1 ng/μL rVpr at 4°C overnight. Then, 100 μL of 1:100 diluted serum was applied and incubated at 37°C for 1 h. After washing with PBS, the plates were incubated with 1:10,000-diluted anti-human IgG antibody conjugated to horseradish peroxidase (GeneTex, Irvine, CA) and developed with TMB substrate solution (Nacalai Tesque, Kyoto, Japan). Absorbance was measured at a wavelength of 450 nm (OD450) using a microplate reader (Bio-Rad, Irvine, CA). HIV-uninfected volunteer samples were used as the negative control, whereas those of infected patients with high levels of anti-Vpr antibodies were used as the positive control. All measurements were made in triplicate. The positive cut-off value was set to the mean plus three times standard deviation of the negative control.

### Inflammatory cytokine ELISA

Inflammatory cytokines, IL-6, TNFα and IL-1β, were measured using the AlphaLISA immune assay kit (PerkinElmer, Waltham, MA) according to the manufacturer’s kit protocols. Briefly, a 384-well plate was filled with 2μl of serum sample, 4μl of 50 μl/mL AlphaLISA acceptor bead-conjugated antibody, and 4μl of 5 nM biotinylated antibody against a cytokine in AlphaLISA immunoassay buffer, then incubated for 1 h at 23°C. Without a washing step, 10 μL of 80 μg/mL streptavidin-coated donor beads were added. After incubation for 30 min at 23°C in the dark, the plate was read on a Nivo microplate reader (PerkinElmer) using the AlphaLISA measurement protocol (excitation wavelength of 680 nm and emission detection to 615 nm). The concentration of cytokines was calculated from the slope of the standard curve of an analyte standard provided by the supplier (PerkinElmer). Lowest limits of quantification (LLOQ) by the AlphaLISA were 1.3 pg/mL of IL-6, 2.2 pg/mL of TNFα, and 0.65 pg/mL of IL-1β, respectively. If the measurement value were below the LLOQ, we used the half values of LLOQ, 0.65 pg/mL for IL-6, 1.1 pg/mL for TNFα, and 0.325 pg/mL for IL-1b, for statistical analyses.

### Statistical analysis

To avoid confounding differences due to baseline varieties in comparing between the non-tumor group and the tumor group, we performed a propensity score matching by SPSS software (version 26; IBM, Chicago, IL). The propensity scores were generated with the prospective characteristics, including age, sex at birth, CD4+ T-cell count, CD8+ T-cell count, HIV viremia, HBV viremia and HCV viremia by fitting a logistic regression model. The matching was performed using 1:1 ratio without replacement by caliper-matching on the estimated propensity score. The caliper was calculated by 0.25 × (standard deviation of propensity score). Other statistical analyses were performed with Prism software (version 9; GraphPad Software, La Jolla, CA). The statistical significance of differences between the two groups was tested using the Wilcoxon matched pairs signed rank test for the matched pair group analysis and the Mann–Whitney U nonparametric test. The differences between categorical variables were analyzed using Fisher’s exact test. Statistical significance was set at *P* < 0.05. To analyze the distribution of Vpr concentration in the tumor and non-tumor groups, we used 1.95 ng/mL for the samples, the concentrations of which were less than 3.9 ng/mL, the detection limit of the ELISA. To compare three groups, a nonparametric Kruskal-Wallis test was performed, and then the post hoc Dunn’s multiple comparisons test was used to identify which specific groups were significantly different from each other. To extract predictor variables with correlation with the outcome variable (IL-6 or Vpr), the multivariate linear regression analyses were performed with forced entry method. Binary logistic regression model was used to identify risk factors associated with tumor occurrence in HIV patients as the reference (odds ratio = 1). Receiver operating characteristic (ROC) curve was plotted to evaluate the performance of risk factors and the area under curve (AUC) was measured to represent the degree of separability.

## Results

### Characteristics of the enrolled HIV patients

The cohort analyzed included 404 HIV patients: 126 patients with malignancies (tumor group) and 278 patients without malignancies (non-tumor group). There was no difference in gender or age distribution between these two groups (*P* > 0.999 and 0.289, respectively; Table 1). Of note, there was a higher frequency of HIV viremia in the tumor group compared with the non-tumor group (37.3% vs. 9.0%, *P* < 0.001; Table 1), which was associated with a significant difference in CD4+ T-cell counts (median [interquartile range], 369 [158.3–543.5] cells/μL for the tumor group vs. 549 [398.5–717.5] cells/μL for the non-tumor group, *P* < 0.001; Table 1) and CD4/CD8 ratios (0.502 [0.201–0929] vs. 0.770 [0.561–1.144], respectively, *P* < 0.001; Table 1). To reduce these biases of confounding factors with significant differences, we extracted 96 patients with malignancies (matched tumor group) and 96 patients without malignancies (matched non-tumor group) by one-to-one propensity score matching. There was no difference in demographic and clinical characteristics between these two matched groups (Table 1).

### Higher detection frequency of Vpr in the tumor group

**Fig 1:**
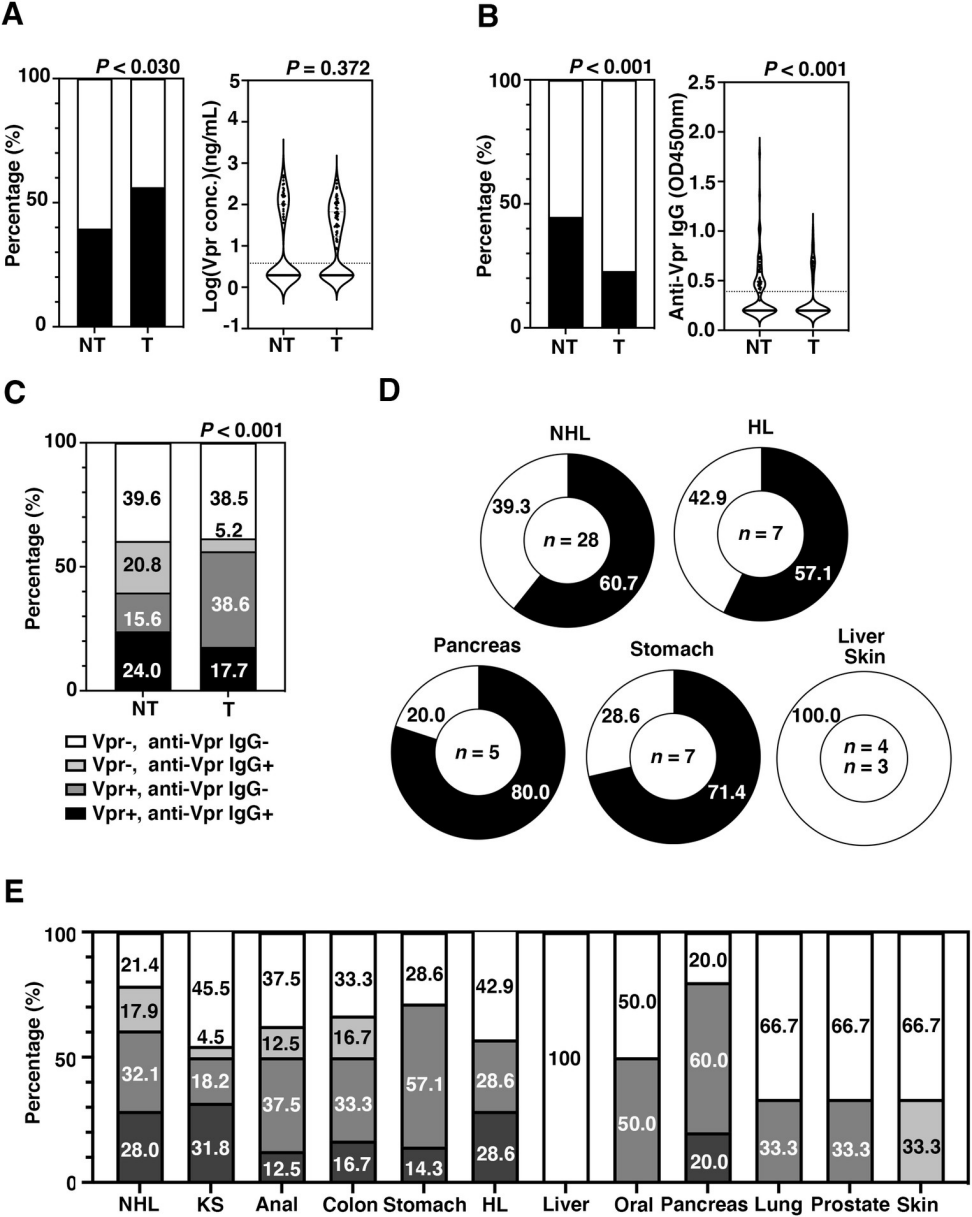
Inverse detection frequencies of circulating Vpr and anti-Vpr IgG in HIV patients with tumors. (A) Incidence of Vpr and distribution of Vpr concentrations in HIV patients. Left panel, stacked bar chart showing the proportion of Vpr-positive samples in the matched tumor group (T) and the matched non-tumor group (NT). Black bar, Vpr positivity. White bar, Vpr negativity. Right panel. violin plot showing the distribution of Vpr concentrations (ng/mL). Dotted line is the detection limit of Vpr (3.8 ng/mL). (B) Incidence of anti-Vpr IgG in HIV patients. Left panel, bar chart showing the proportion of anti-Vpr IgG-positive samples in HIV patients. Black bar, anti-Vpr IgG positivity. White bar, anti-Vpr IgG negativity. Right panel, violin plot showing the distribution of anti-Vpr IgG titers (OD value). Dotted line shows the value of the negative control (OD = 0.38). (C) Combined positive proportion of Vpr and anti-Vpr IgG. The proportion of Vpr and anti-Vpr IgG-double positive (Vpr+, anti-Vpr IgG+; dark ray bar), only Vpr-positive (Vpr+, anti-Vpr IgG-; gray bar), only anti-Vpr IgG-positive (Vpr-, anti-Vpr IgG+; light gray bar), and Vpr and anti-Vpr IgG-double negative (Vpr-, anti-Vpr IgG-, white bar) patients was depicted. The percentage of each group was shown with a white or black number. Fisher’s exact test (for 2×2 contingency table) or chi-square test (for 2×4 contingency table) was used to compare the number of patients between the T and NT groups. (D) Relative frequency of cases positive for Vpr in representative tumors Representative doughnut chart depicting the relative frequency of Vpr-positive (black) and -negative (white) patients with NHL, HL, pancreatic cancer, stomach cancer, liver cancer, and skin cancer. The percentage of Vpr-positive and -negative patients was shown in white or black numbers, respectively. (E) The proportion of combined detection of Vpr and anti-Vpr IgG in each primary lesion of tumor. The percentage of each group was shown with a white or black number.

In 96 patients of the matched tumor group, we detected 54 patients positive for circulating Vpr (56.3%), whereas 38 of 96 patients were positive in the matched non-tumor group (39.6%). Data implied that Vpr is present at significantly higher frequency in the tumor group than in the non-tumor group (*P* = 0.0300; Fig 1A, left panel). However, there was no remarkable difference in the concentrations of Vpr between the two groups (*P* = 0.372; Fig 1A, right panel). The clinical characteristics were also similar in the Vpr-positive and -negative cases (Table 2). Additionally, both Vpr detection frequency and concentration distribution were not different in the aviremic (HIV RNA level < detection limit 20 copy/mL) and viremic patients among both the non-tumor group and the tumor-group (*P* = 0.672 and *P* = 0.769 for the non-tumor group and *P* = 0.716 and *P* = 0.309 for the tumor group; S1A Fig). The incidence of viral blips in ART-controlled patients, in which the viral load temporarily increased to a maximum of 500 copies/mL, but quickly decreased to <50 copies/mL, was 5.5–8.3% and not different between two groups (*P* = 0.855 for the tumor group and *P* = 0.744 for the non-tumor group; Table 2). Data also indicated that circulating Vpr is not directly linked with the HIV present in the peripheral blood. Moreover, the detection frequency of Vpr was not different in NADM and ADMs (*P* = 0.469; S1B Fig and S1 Table) or the virus-related (VR) and the virus-unrelated (VUR) tumors (*P* = 0.597 for VR and VUR; S1C Fig).

**Table 2:** Relationships between circulating Vpr and clinical characteristics.

Group of HIV patients	Tumor			Non-tumor		
Vpr	Positive	Negative	*P*-value	Positive	Negative	*P*-value
	(*n* = 66, 52.4%)	(*n* = 60, 47.6%)		(*n* = 115, 41.4%)	(*n* = 163, 58.6%)	
Sex at birth						
Female	2 (3.0)	5 (8.3)	0.256	8 (7.0)	7 (4.3)	0.421
Male	64 (97.0)	55 (91.7)		107 (93.0)	156 (95.7)	
Age (years)	51.5 [42.75–64]	53 [43–64]	0.809	50 [46–56]	49 [45–56]	0.817
CD4+ T-cells (cells/μL)	394 [196.8–549.8]	279 [134–539.3]	0.260	560 [394–739]	541 [407–682]	0.590
CD8+ T-cells (cells/μL)	640.5 [446–1006]	625 [471–860]	0.804	700 [559–933]	699 [497–926]	0.398
CD4/CD8 ratio	0.536 [0.222–0.927]	0.455 [0.150–0.929]	0.496	0.802 [0.534–1.155]	0.758 [0.571–1.116]	0.675
CRP (mg/dL)	0.30 [0.10–1.13]	0.20 [0.10–0.60]	0.0853	0.08 [0.04–0.19]	0.06 [0.03–0.12]	0.0358
Viremia						
HIV	24 (36.4)	24 (40.0)	0.716	9 (7.8)	16 (9.8)	0.672
Viral blip	4 (6.1)	5 (8.3)	0.855	6 (5.2)	9 (5.5)	0.744
HBV	6 (9.4)	10 (16.9)	0.285	7 (6.2)	11 (6.7)	>0.999
HCV	7 (10.6)	7 (11.7)	>0.999	2 (1.8)	5 (3.1)	0.704
ART	57 (86.4)	48 (80.0)	0.352	115 (100.0)	163 (100.0)	>0.999

Data are *n* (%) or median [interquartile range].

Fisher’s exact test was to look for differences in categorical variables.

Mann–Whitney *U* test was conducted as a test of significance for continuous variables.

### Lower detection frequency of anti-Vpr IgG in the tumor group

Given that some HIV-infected patients had seroconversion against Vpr [49–52], we next measured anti-Vpr IgG. Data revealed that in the tumor group, 22 patients (22.9%) were positive for anti-Vpr IgG, whereas 43 patients (44.8%) were positive in the non-tumor group, suggesting that the detection frequency of anti-Vpr IgG was significantly lower in the matched tumor group than in the matched non-tumor group (*P* = 0.0022; Fig 1B left panel), although the distribution of the anti-Vpr IgG concentration was significantly higher in the matched non-tumor group (*P* < 0.0001; Fig 1B, right panel). Curiously, the detection frequency of anti-Vpr IgG level was higher in the viremic patients of the tumor group (61.8% vs. 29.3%, *P* = 0.0017; Table 3) who had a lower CD4+ T-cell count (246 [121–404] vs. 409 [173–602], *P* = 0.0123; Table 3) and a lower CD4/CD8 ratio (0.322 [0.149–0.609] vs. 0.596 [0.286–1.062], *P* = 0.0021; Table 3). The anti-Vpr IgG level was not correlated with RNA level of HIV-1 (*P* = 0.700; S2A Fig), and the relationship between anti-Vpr IgG and HIV viremia remains unknown. No positive correlation of Vpr concentrations and anti-Vpr IgG level was observed both in non-tumor and tumor-groups (*P* = 0.812 for the non-tumor group, and *P* = 0.615 for the tumor group; S2B Fig).

**Table 3:** Relationships between anti-Vpr IgG and clinical characteristics.

Group of HIV patients	Tumor			Non-tumor		
Anti-Vpr IgG	Positive	Negative	*P*-value	Positive	Negative	*P*-value
	(*n* = 34, 27.0%)	(*n* = 92, 73.0%)		(*n* = 108, 38.8%)	(*n* = 170, 61.2%)	
Sex at birth						
Female	0 (0.0)	7 (7.6)	0.188	8 (7.4)	7 (4.1)	0.280
Male	34 (100.0)	85 (92.4)		100 (92.6)	163 (95.9)	
Age (years)	47 [41–55]	55 [44–65]	0.0525	49 [46–54.8]	50 [45–59]	0.227
CD4+ T-cells (cells/μL)	246 [121–404]	409 [173–602]	0.0123	525 [394.5–705.5]	599 [405–719.3]	0.391
CD8+ T-cells (cells/μL)	653.5 [497–929.3]	621 [434.5–886]	0.113	699 [527–871.8]	702.5 [525–937.5]	0.901
CD4/CD8 ratio	0.322 [0.149–0.609]	0.596 [0.286–1.062]	0.0021	0.760 [0.545–1.080]	0.821 [0.564–1.173]	0.378
CRP (mg/dL)	0.20 [0.10–0.75]	0.20 [0.10–1.08]	0.794	0.05 [0.02–0.10]	0.06 [0.03–0.14]	0.0777
Viremia						
HIV	21 (61.8)	27 (29.3)	0.0017	12 (11.1)	13 (7.6)	0.391
HBV	5 (15.2)	11 (12.2)	0.763	6 (5.6)	12 (7.1)	0.804
HCV	1 (2.9)	13 (14.1)	0.110	7 (6.6)	0 (0.0)	0.0011
ART	26 (76.5)	79 (85.9)	0.280	108 (100.0)	170 (100.0)	>0.999

Data are *n* (%) or median [interquartile range].

Fisher’s exact test was used to look for differences in categorical variables.

Mann–Whitney *U* test was conducted as a test of significance for continuous variables.

We next analyzed the correlation of Vpr and anti-Vpr IgG in relation to tumor occurrence. As shown in Fig 1C, the detection frequency of Vpr in the anti-Vpr IgG-negative patients was significantly higher in the tumor group compared with the non-tumor group (38.6% vs. 15.6%, respectively, *P* < 0.001). In striking contrast, the detection frequency of anti-Vpr IgG in the Vpr-negative patients was significantly higher in the non-tumor group compared with the tumor group (20.8%% vs. 5.2%, respectively; Fig 1C). Data suggest that anti-Vpr IgG functions as a negative factor for tumorigenesis counteracting Vpr activity.

Quite interestingly, Vpr was detected at high frequency in particular types of tumors such as NHL (60.7% patients for Vpr positive), HL (57.1%), stomach (71.4%), and pancreatic cancers (80.0%) whereas it was not detected in live and skin cancers (0.0%; Fig 1D). The proportion of the patients who were positive for Vpr but negative of anti-Vpr IgG-negative was especially high in the stomach (57.1%), oral (50.0%), and pancreas cancers (60.0%) (Fig 1E), implicating the possible involvement of Vpr in certain types of tumor development.

### Positive link of Vpr and IL-6 under non-tumor conditions

**Fig 2:**
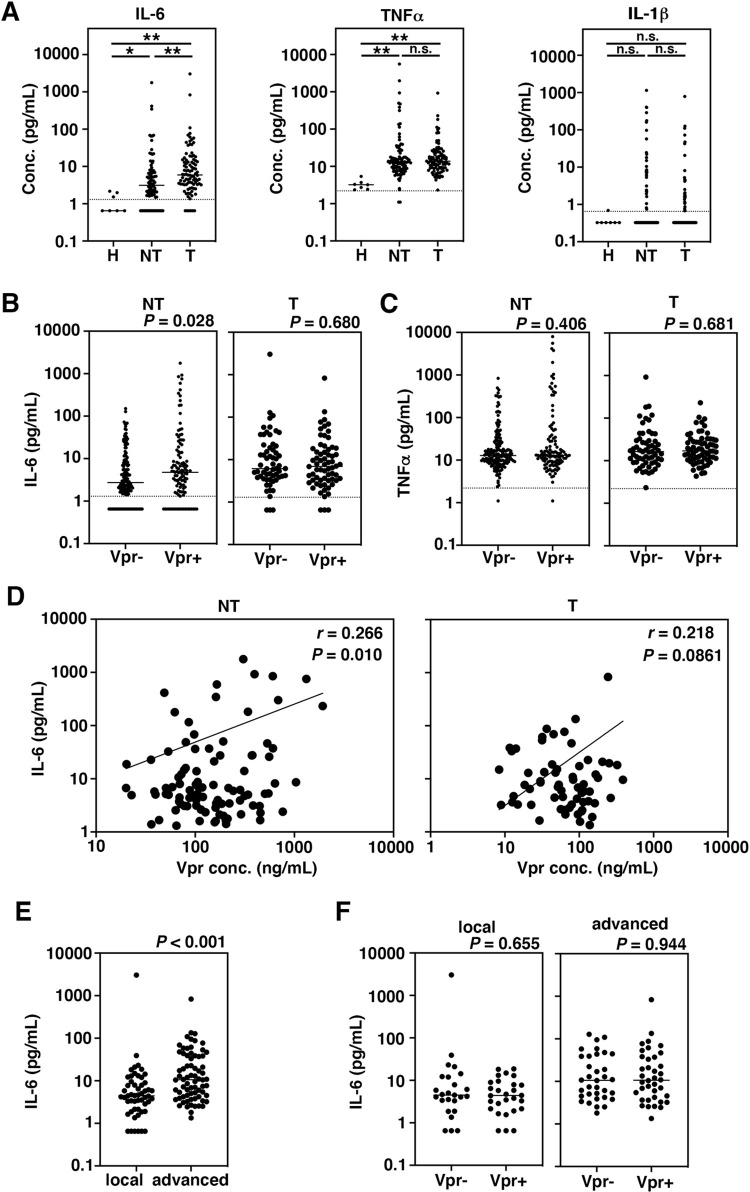
Circulating Vpr and inflammatory cytokines. (A) Detection of cytokines. Concentrations of IL-6, TNFα and IL-1β detected in the patients of the matched tumor and the matched non-tumor groups were plotted (pg/mL). H, non-HIV infected healthy individuals; NT, HIV patients with non-tumor; T, HIV patients with tumor. Dotted line is the lower quantification limit (LLOQ) of IL-6 (1.3 pg/mL), of TNFα (2.2 pg/mL) and of IL-1b (0.65 pg/mL). Samples below LLOQ were plotted at half the detection limit (0.65 ng/mL for IL6, 1.1 pg/mL for TNFα, and 0.325 pg/mL for IL-1β). Horizontal bar represents the median value. *P < 0.05, **P < 0.001. (B) High concentrations of IL-6 in the Vpr-positive patients in the tumor group. Concentrations of IL-6 in the Vpr-negative (Vpr-) and Vpr-positive (Vpr+) groups in NT and T patients were plotted. Dotted line is the LLOQ of IL-6 (1.3 pg/mL). (C) TNFα concentration in the Vpr-negative (Vpr-) and Vpr-positive (Vpr+) groups in NT and T patients. Dotted line is the LLOQ of TNFα (2.2 pg/mL). (D) Correlation between Vpr and IL-6 concentrations in two groups. A log-log regression analysis was performed to assess the correlation. The correlation coefficient (r) and the p-value (*P*) are shown in graph. The solid line represents the best-fit line. In the T group, concentrations of IL-6 tended to be high in the Vpr-positive patients, but their statistical correlation was not observed. (E) IL-6 concentration in both local and advanced cases of HIV patients with malignancies. (F) IL-6 concentration in Vpr- and Vpr+ groups of local and advanced malignancies.

We measured IL-6, TNFα, and IL-1β, which are risk factors for cancer development [53] and are also involved in HIV-1 pathogenesis [54]. We found that concentrations of both IL-6 and TNFα were significantly high in the matched non-tumor and the matched tumor groups than healthy people (H) (*P* = 0.0289 and *P* < 0.001 for IL-6, and *P* = 0.001 and *P* < 0.001 for TNFα, respectively; Fig 2A), whereas IL-1β was not (*P* = 0.7156 and *P* > 0.9999; Fig 2A). Although the TNFα level was not different in the tumor and the non-tumor groups (*P* > 0.9999), the IL-6 level was significantly high in the tumor group (*P* < 0.001; Fig 2A, left panel). Further analysis revealed that IL-6 level was significantly higher in the non-tumor group of patients who were positive for Vpr (*P* = 0.0283; Fig 2B, left panel), but not in the tumor group (*P* = 0.680; Fig 2B, right panel). This observation was unique for IL-6, because there was no significant difference of TNFα in patients with or without Vpr among non-tumor and the tumor groups (*P* = 0.406 and *P* = 0.681, respectively; Fig 2C). To identify factors that correlate with IL-6, we performed the linear regression analyses in the non-tumor group and the tumor group and observed that Vpr concentration was the single molecule that correlated with IL-6 in the non-tumor group (S2 Table). We plotted the concentrations of Vpr and IL-6 and found that these molecules are well correlated in the non-tumor group (*r* = 0.266, *P* = 0.0095; Fig 2D, left panel). In the tumor group, however, there was no positive correlation between these molecules (*r* = 0.218, *P* = 0.0861; Fig 2D, right panel). Well consistent with the report that serum IL-6 is a risk factor for tumor progression [55], we observed that IL-6 concentrations were significantly high in the patients with advanced tumors (*P* < 0.001; Fig 2E), but it was not linked with Vpr (*P* = 0.655 for local tumor, and *P* = 0.944 for systemic tumor; Fig 2F).

### Vpr as a novel risk factor for tumor in HIV patients

**Fig 3:**
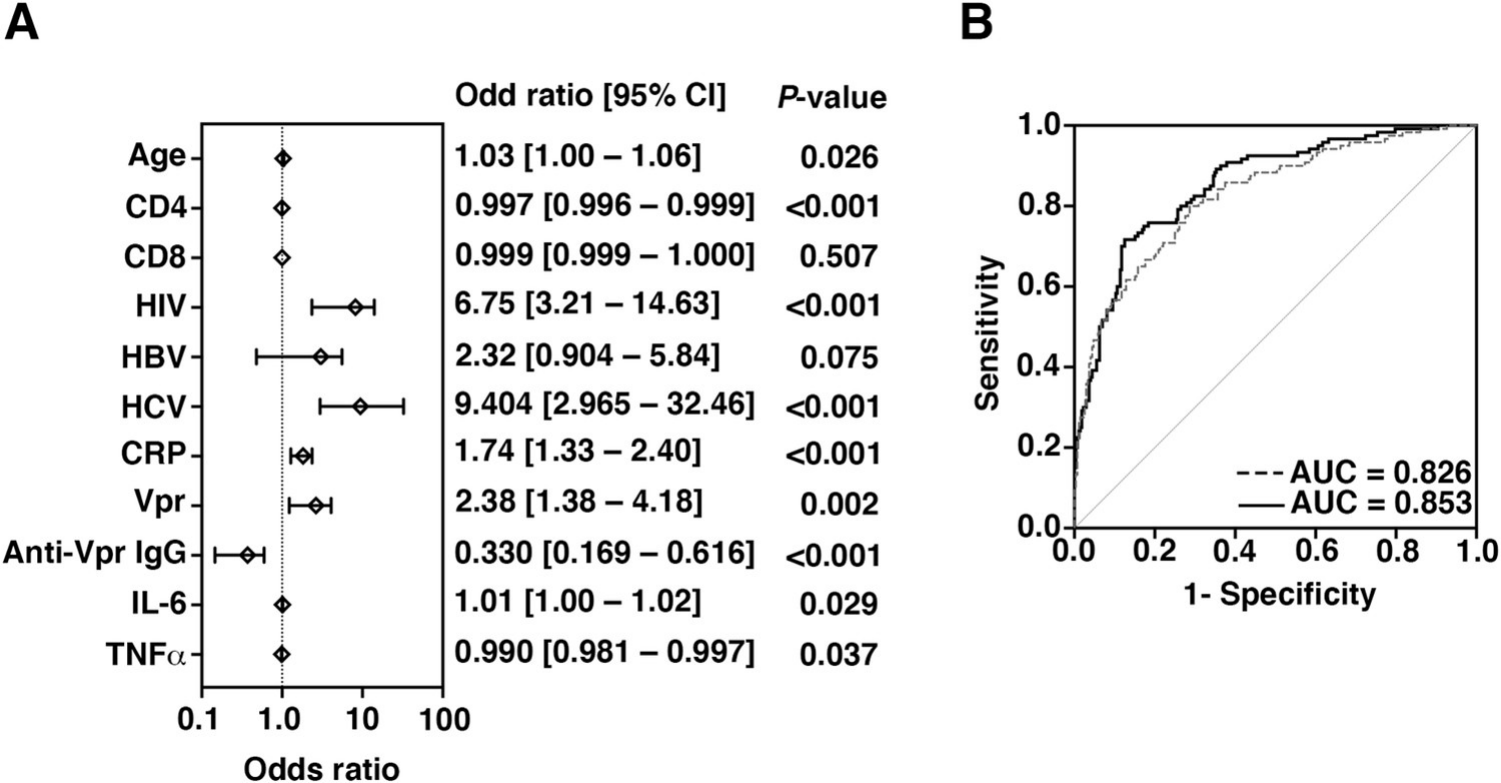
Risk factors associated to tumor occurrence in HIV patients. (A) Forest plot of odds ratio by multivariate logistic regression analysis. (B) ROC curves. Dashed line represents the performance on risk factors, age, CD4, CD8, HIV, HBV, HCV, CRP, IL-6 and TNFα, without Vpr and anti-Vpr IgG. Solid line represents the performance on risk factors including Vpr and anti-Vpr IgG. AUC, area under the curve.

To analyze the risk factors for tumor occurrence in HIV patients, we finally performed multiple logistic regression analysis. The result showed that Vpr was positively related with tumor occurrence (odds ratio [85% confidence interval] = 2.38 [1.38–4.18]; *P* = 0.002; Fig 3A) whereas anti-Vpr IgG was negatively related with that (0.330 [0.169–0.616]; *P* < 0.001; Fig 3A). Beside Vpr, age, HIV viremia, HCV viremia, CRP, and IL-6 were also related with tumor occurrence. Furthermore, the classification between the non-tumor and the tumor groups could be more correctly predicted by including information of Vpr and anti-Vpr IgG into age, CD4, CD8, HIV viremia, HBV, viremia, HCV viremia, IL-6, and TNFα. (AUC = 0.853, and AUC = 0.826, respectively; Fig 3B). These data suggested that Vpr was a novel risk factor for tumorigenesis in HIV patients.

## Discussion

Here we found that the circulating Vpr is present at a significantly high frequency in the tumor group compared with the non-tumor group (Fig 1A and Table 2). It is particularly interesting that Vpr was highly positive in NHL, HL, pancreatic, and stomach cancers (Fig 1D) and that the detection frequency of anti-Vpr IgG was low in the patients with gastric and pancreatic cancers (Fig 1E). Lines of evidence indicate that serum Vpr is biologically active [31], and extracellular Vpr causes mitochondrial stress, DNA damage, and retrotransposition at the ten ng/mL range [39,41,44]. Intriguingly, the median concentration of Vpr in HIV-positive patients was 152.5 ng/mL, implicating that approximately 750 μg of Vpr (0.15 μg/mL x 5000 mL total blood) can be continuously produced *in vivo* and the concentration in the vicinity of the infected cells would be high enough for inducing cellular damage. Because pancreatic acinar cells are the target of HIV infection via C-C chemokine receptor 5, both intracellular and secreted Vpr can induce mitochondrial stress and DNA damage on not only the infected acinar cells but also adjacent cells as a bystander effect [56,57]. If anti-Vpr IgG is not present, a high concentration of Vpr possibly induces more advanced pancreatitis and genotoxic insults leading to cancer development.

It has been proposed that even under ART control, chronic inflammation is continuously observed in HIV patients and plays a risk factor for malignancies [47,58]. Because Vpr is responsive for the induction of multiple inflammatory cytokines such as IL-1β [59], IL-6 [42,43], and TNF-α [46], we here measured these cytokines and investigated the possible link with Vpr. As shown in Fig 2A, the levels of IL-6 and TNFα were higher in HIV-positive patients compared with non-HIV infected healthy individuals, consistent with previous reports [60,61]. Although it has been reported that higher levels of IL-6 in plasma were associated with older age, higher HIV RNA, lower nadir CD4+ T-cell count and others during HIV infection [62], our current analysis revealed that IL-6 was correlated only with Vpr especially in the non-tumor group but not with age, HIV viremia and CD4+ T-cell count (*P* < 0.001; Fig 2D, left panel and S2 Table). The level of IL-6 was significantly higher in the patients with advanced tumors than local tumors (Fig 2E), but was not statistically correlated with the concentration of Vpr, or the presence of Vpr (Fig 2F). Data suggest that under non-tumor conditions, IL-6 concentration is directly influenced by Vpr, but once tumors are developed, IL-6 is produced by multiple factors alongside Vpr, for example, inflammation or tissue damage that associated with tumor development [63]. Because chronic overproduction of IL-6 can stimulate cell proliferation and survival [64], it is likely that Vpr and IL-6 are cooperatively involved in cellular transformation during tumorigenesis.

As to the emergence of viral proteins in patients’ blood, two possibilities have been postulated. First, there is evidence for residual viral replication due to the low concentrations of antiretroviral drugs in sanctuary sites are present throughout the body, including the lymph nodes, central nervous system, tissue macrophages, adipose tissue, and the gut-associated lymphoid tissue [65–70]. It has also been reported that temporal elevation of viral production, “viral blip”, is induced by transient inflammation in the sanctuary sites [71,72]. In our study, the proportion of viral blips in the Vpr-positive patients was negligible both in the tumor group (5.2%, 6 of 115 patients) and the non-tumor group (6.1%, 4 of 66 patients) (Table 3) and none of factors including HIV viremia were predicted with Vpr emergence status (S3 Table), further implying that the production of Vpr was not due to the viral blip. Second, reservoir cells with a defective provirus genome survive and secrete viral proteins for long periods. Under ART control, the vast majority (approximately 95%) of HIV proviruses, which are integrated into the CD4+ T-cell genome, are defective with large internal deletions, premature stop codons, and G-to-A hypermutations [73]. Even though these proviruses are unable to produce replication-competent viruses [74], they can generate fragments of HIV transcripts and produce encoded viral proteins [75]. Toward complete cure of HIV, various trials that include shock-and kill strategy and block-and-lock strategy have been recently designed and tried. In the shock-and kill strategy, it is expected that the reactivated virus from the cell reservoir is eliminated by the immune system or cytopathic effects [76,77]. However, cells with large internal deletion of the provirus genome would escape from HIV-specific cytotoxic T lymphocytes [73], implying that the block-and-lock strategy, in which transcription from the provirus genome is permanently shut off by inducing a deep and irreversible latency is more hopeful [78]. In any challenge, it is important to evaluate the efficacy of the system, and Vpr-ELISA would be useful for monitoring.

As potent Vpr inhibitors, several small molecules have been reported. They include vipirinin, fumagillin, quercetin, and VTD227, which inhibited Vpr-induced cell cycle arrest (G2/M arrest) and viral infection of macrophages [79–82]. They also include damnacanthal, isopimarane diterpenoids, icrasane quassinoids, bis-iridoid and iridoid glycosides, which inhibited Vpr-induced cell death [83–86]. Another inhibitor is mifepristone, which inhibited viral replication via extracellular Vpr and glucocorticoid receptor (GR) mediated HIV-1 LTR transactivation [87,88]. The effect of these Vpr inhibitors on the Vpr-induced IL-6 production has not been reported yet. Regarding the IL-6 promoter activation, glucocorticoids have both antagonistic and agonistic functions through five putative GR binding sites [89]. If the Vpr–GR complex could induce the IL-6 production via TLR4/MyD88 signaling [42], mifepristone might be a potential drug for preventing tumorigenesis caused by Vpr in HIV patients. The nuclear localization of Vpr–GR and poly (ADP-ribose) polymerase-1 complex was inhibited by anti-Vpr monoclonal antibody [90]. We previously reported that extracellular Vpr-induced retrotransposition activity was inhibited by anti-Vpr monoclonal antibody [44], implicating that anti-Vpr antibody drugs might be also useful for preventing Vpr-induced tumorigenesis in HIV patients.

Here we focused on Vpr, but other viral proteins such as Tat, Nef, gp120, and p17 have been reported to be present in patients’ blood under ART [13–17]. Of these viral proteins, Tat is involved in the transcriptional activation of host cell genes such as oncogenic *c-MYC* and activation-induced cytidine deaminase in B-cells, leading to B-cell malignancies [91,92]. HIV-1 p17, a structural matrix protein, promotes angiogenesis and lymphangiogenesis [93,94]. Its variant stimulates B-cell proliferation and is enriched in patients with NHL [95]. Vpr may co-operates with Tat or p17 for the development of B-cell malignancies because Vpr can bind directly to Tat or p17 and function in viral gene transcription and maturation [96,97]. It would be important to monitor these viral proteins by combining with Vpr and IL-6, since the current study revealed that these two molecules are candidate risk factors for tumorigenesis.

Our study had some limitations. In Vpr-ELISA, samples are subjected to analysis without use of any detergents [45], indicating that only soluble Vpr present as a free-form in blood is detected. This may raise the possibility that Vpr, which is masked by anti-Vpr IgG, might not be measured accurately. However, the detection frequency of Vpr in the non-tumor group was 39.6% (Fig 1A), well consistent with our previous report that 6 of 15 samples of patient serum were active in inducing retrotransposition [44]. Data imply that the frequency of biologically active Vpr was successfully detected by Vpr-ELISA. Second, we here measured Vpr in a single sample of each patient, but it would be necessary to examine several samples that are collected at different time points. It is especially important to know whether Vpr is continuously detected in the same patients, or not. By analyzing a larger cohort of HIV patients at different time-points, more reliable data would help us understand the roles of Vpr in tumor development and detecting malignancies at an early stage.

## Supporting information

S1 Checklist STROBE statement–checklist of items that should be included in reports of cohort studies. (DOCX)

S1 Fig Circulating Vpr under HIV viremic conditions and oncovirus-related malignancies. (A) Stacked bar chart showing the proportion of Vpr-positive samples in HIV patients with aviremia (Av) and viremia (V). Black bar, Vpr positivity. White bar, Vpr negativity. Violin plot showing the distribution of Vpr concentrations (ng/mL). Dotted line is the detection limit of Vpr (3.8 ng/mL). (B) The detection frequency of Vpr in patients with AIDS-defining malignancies (ADM) and non-AIDS defining malignancies (NADM). (C) The detection frequency of Vpr in patients with virus-related (VR) and virus-unrelated malignancies (VUR).
(PDF)

S2 Fig Correlation of anti-Vpr IgG level, HIV RNA level and Vpr levels. (A) Relationship between anti-Vpr IgG level and HIV RNA in 72 HIV viremic patients. The horizontal dotted line is the detection limit of HIV RNA (20 copies/mL), and the vertical dotted line is the detection limit of anti-Vpr IgG (optical density at 450nm; OD450 = 0.38). (B) Relationship between anti-Vpr IgG and Vpr levels in the non-tumor and tumor groups. The horizontal dotted line is the detection limit of Vpr (3.9 pg/mL), and the vertical dotted line is the detection limit of anti-Vpr IgG (OD = 0.38). A log-log regression analysis was performed to assess the correlation. The correlation coefficient (*r*) and the p-value (*P*) are shown in graph. The solid line represents the best-fit line.
(PDF)

S1 Table Clinical characteristics of HIV patients with ADM and NADM. (PDF)

S2 Table Multiple linear regression analysis of association between factors and IL-6 concentration. (PDF)

S3 Table Multiple logistic regression analysis of association between factors and Vpr expression. (PDF)
