# Supplementary figures and images for: Identification of viral protein R of human immunodeficiency virus-1 (HIV) and interleukin-6 as risk factors for malignancies in HIV-infected individuals: A cohort study
Source: PLoS One. 2024 Jan 2;19(1):e0296502. doi: 10.1371/journal.pone.0296502 (PMC10760899; doi:10.1371/journal.pone.0296502)

S1 Fig.

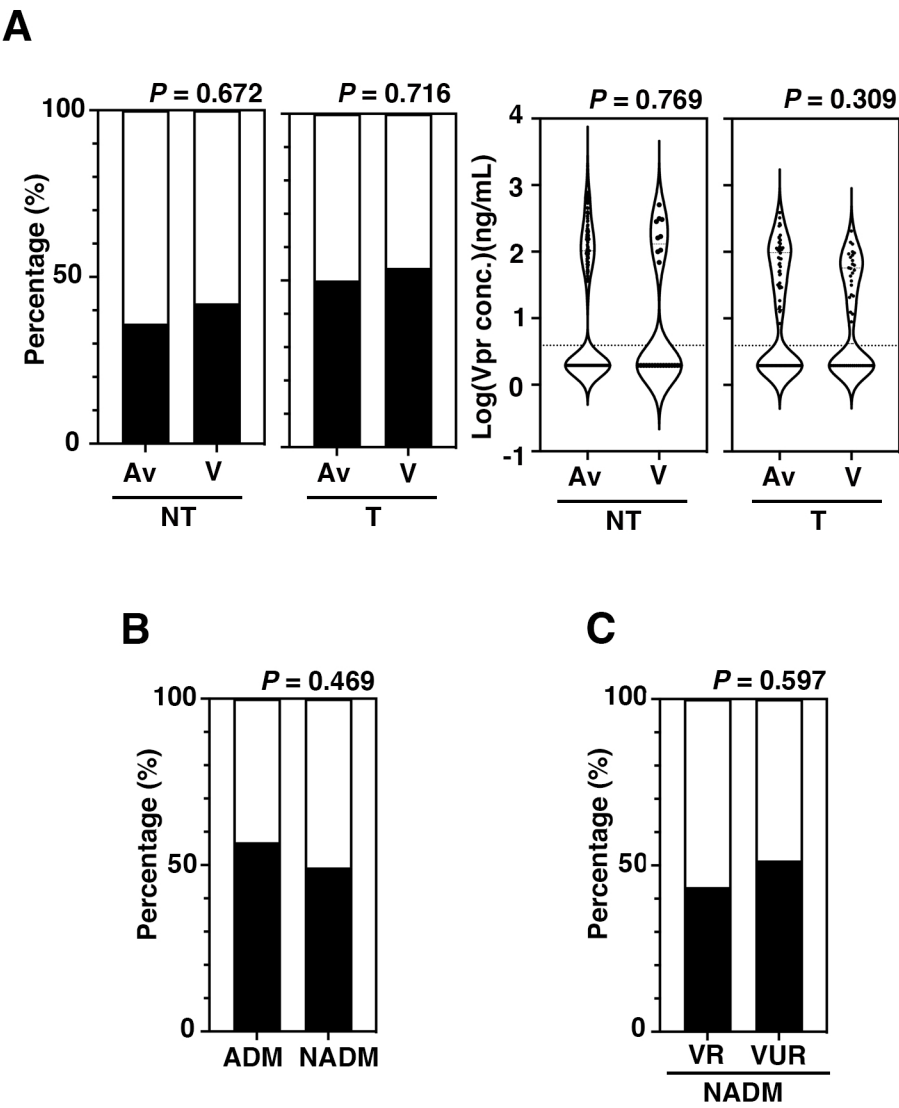

Supplement: S1 Fig — (A) Stacked bar chart showing the proportion of Vpr-positive samples in HIV patients with aviremia (Av) and viremia (V). Black bar, Vpr positivity. White bar, Vpr negativity. Violin plot showing the distribution of Vpr concentrations (ng/mL). Dotted line is the detection limit of Vpr (3.8 ng/mL). (B) The detection frequency of Vpr in patients with AIDS-defining malignancies (ADM) and non-AIDS defining malignancies (NADM). (C) The detection frequency of Vpr in patients with virus-related (VR) and virus-unrelated malignancies (VUR). (PDF) [file pone.0296502.s002.pdf]

S2 Fig.

A

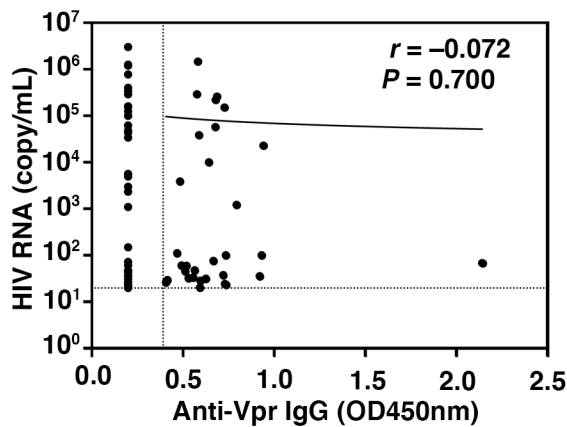

B

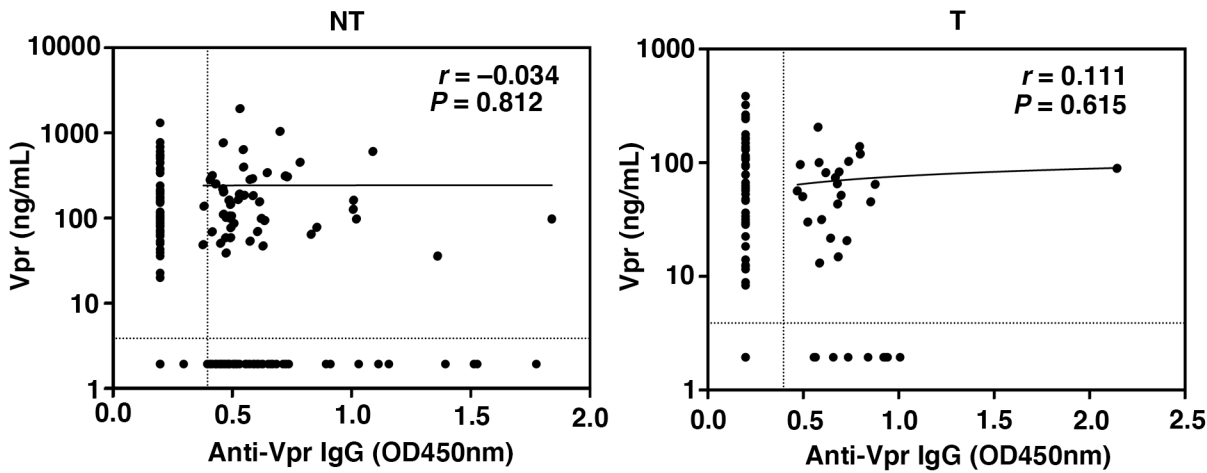

Supplement: S2 Fig — (A) Relationship between anti-Vpr IgG level and HIV RNA in 72 HIV viremic patients. The horizontal dotted line is the detection limit of HIV RNA (20 copies/mL), and the vertical dotted line is the detection limit of anti-Vpr IgG (optical density at 450nm; OD450 = 0.38). (B) Relationship between anti-Vpr IgG and Vpr levels in the non-tumor and tumor groups. The horizontal dotted line is the detection limit of Vpr (3.9 pg/mL), and the vertical dotted line is the detection limit of anti-Vpr IgG (OD = 0.38). A log-log regression analysis was performed to assess the correlation. The correlation coefficient (r) and the p-value (P) are shown in graph. The solid line represents the best-fit line. (PDF) [file pone.0296502.s003.pdf]
