# Supplementary material for: Identification of viral protein R of human immunodeficiency virus-1 (HIV) and interleukin-6 as risk factors for malignancies in HIV-infected individuals: A cohort study
Source: PLoS One. 2024 Jan 2;19(1):e0296502. doi: 10.1371/journal.pone.0296502 (PMC10760899; doi:10.1371/journal.pone.0296502)
Supplement: S1 Table — (PDF) [file pone.0296502.s004.pdf]

S1 table. Clinical characteristics of HIV patients with ADM and NADM

Patients	ADM<br>(*n* = 51)	NADM<br>(*n* = 75)	*P*-value
Sex at birth			
Female	2 (3.9)	5 (6.7)	0.700
Male	49 (96.1)	70 (93.3)	
Age (years)	45 [39 – 54]	57 [47 – 66]	<0.001
CD4+ T-cells (cells/µL)	217.0 [84.0 – 394.0]	459.0 [238.0 – 634.0]	<0.001
CD8+ T-cells (cells/µL)	762.5 [474.8 – 1180]	607.0 [447.0 – 829.0]	0.0296
CD4/CD8 ratio	0.229 [0.126 – 0.463]	0.721 [0.419 – 1.085]	<0.001
CRP (mg/dL)	0.30 [0.10 – 0.83]	0.20 [0.10 – 1.20]	0.757
Viremia			
HIV	35 (68.6)	13 (17.3)	<0.001
HBV	7 (14.6)	9 (12.0)	0.785
HCV	0 (0.0)	14 (18.7)	<0.001

Data are *n* (%) or median [interquartile range].

ADM, AIDS-defining malignancy; NADM, non-AIDS-defining malignancy.

Fisher's exact test was used to look for differences in categorical variables.

Mann–Whitney *U* test was conducted as a test of significance for continuous variables.
