# Supplementary material for: Identification of viral protein R of human immunodeficiency virus-1 (HIV) and interleukin-6 as risk factors for malignancies in HIV-infected individuals: A cohort study
Source: PLoS One. 2024 Jan 2;19(1):e0296502. doi: 10.1371/journal.pone.0296502 (PMC10760899; doi:10.1371/journal.pone.0296502)
Supplement: S2 Table — (PDF) [file pone.0296502.s005.pdf]

S2 table. Multiple linear regression analysis of association between factors and IL-6 concentration

	Tumor				Non-tumor			
Variables	Coefficient	SE	t-value	*P*-value	Coefficient	SE	t-value	*P*-value
Sex at birth	59.08	113.9	0.5189	0.6048	25.17	37.35	0.6740	0.5009
Age	-0.0758	2.230	0.0340	0.9729	-0.5854	0.9020	0.6490	0.5169
CD4+ T-cells	0.1507	0.1125	0.9395	0.3494	-0.0149	0.0334	0.4453	0.6564
CD8+ T-cells	0.0097	0.0387	0.2515	0.8018	0.0142	0.0242	0.5864	0.5581
HIV Viremia	-22.07	62.32	0.3541	0.7239	44.77	31.28	1.432	0.1534
Vpr	-0.0322	0.3705	0.0868	0.9310	0.2246	0.0408	5.505	<0.0001

SE, standard error.
