# Supplementary material for: Identification of viral protein R of human immunodeficiency virus-1 (HIV) and interleukin-6 as risk factors for malignancies in HIV-infected individuals: A cohort study
Source: PLoS One. 2024 Jan 2;19(1):e0296502. doi: 10.1371/journal.pone.0296502 (PMC10760899; doi:10.1371/journal.pone.0296502)
Supplement: S3 Table — (PDF) [file pone.0296502.s006.pdf]

S3 table. Multiple logistic regression analysis of association between factors and Vpr expression

	Tumor				Non-tumor			
Variables	Coefficient	SE	t-value	*P*-value	Coefficient	SE	t-value	*P*-value
Sex at birth	0.3517	0.2063	1.705	0.0909	-0.1494	0.1305	1.145	0.2533
Age	-0.0023	0.0039	0.5775	0.5647	0.0013	0.0032	0.4050	0.6858
CD4+ T-cells	0.00024	0.00020	1.190	0.2364	-3.317e-005	0.00011	0.23842	0.7765
CD8+ T-cells	-3.997e-005	7.390e-005	0.5408	0.5897	0.00012	8.474e-005	1.471	0.1424
HIV Viremia	0.0071	0.1125	0.0630	0.9499	-0.1136	0.1099	1.033	0.3024
IL-1β	0.0011	0.0016	0.6764	0.5001	0.00017	0.00019	0.8903	0.3741
IL-6	-0.00018	0.00057	0.3256	0.7453	0.00047	0.00043	1.107	0.2693
TNFα	-0.00087	0.0014	0.6318	0.5287	-7.956e-006	9.564e-005	0.0832	0.9338

SE, standard error.
